# Convulsions from Cocaine Injection

**Published:** 1888-07

**Authors:** A. M. Autrey

**Affiliations:** Houston, Texas


### CORRESPONDENCE.

### CONVULSIONS FROM COCAINE INJECTION.

HOUSTON, TEXAS, June 18, 1888.

*Editor Daniel's Texas Medical Journal:*

Having read in your JOURNAL of this month the query of Dr. R. H. Harrison, Jr., of Columbus, Texas, recalls to my mind an article in "La Tribune Medicale," which will answer it; a communication of M. Richet, of Paris, France, to the "Societe de Biologia" at its meeting of the 5th of May last. It it as follows:

"Epileptiform convulsions supervene upon the subcutaneous injections of cocaine in animals. However, if a section of the spinal cord is practised previously to the injection, the convulsions will not occur. Is it not then likely that cocaine acts through this conducting organ directly upon the encephalon? The experiments which I have made, enable me to state that when the motor centers are destroyed before practising the injection, it requires a much larger dose of cocaine to bring on the convulsions, besides, the form of the convulsions itself is modified. Therefore, judging from the facts, I would suppose that the convulsive effects of cocaine are due to an excitation of the cortical motor centers of the brain."

To this I may add that in the case of Dr. L., referred to by Dr. Harrison, I would attribute the effects noticed to the cocaine, though these are said to have been immediate; for having had frequent occasions to use cocaine in my practice in this city, I have noticed that in those cases in which it was not possible to prevent it from going into the general circulation, due to the part of the body into which it was injected, dilatation of the pupil and some nervousness, though never amounting to convulsions, would sometimes occur before I had had time to finish making the injection. Now supposing these convulsions to be dependent upon the excitation of the nerve center, I would find it reasonable to suppose that no rise in temperature should occur, and no inclination to sleep should be felt after the convulsions had ceased.

A. M. AUTREY, M. D.

